# Role of p53 in breast cancer progression: An insight into p53 targeted therapy

**DOI:** 10.7150/thno.81847

**Published:** 2023-02-27

**Authors:** Charlie Marvalim, Arpita Datta, Soo Chin Lee

**Affiliations:** 1 Cancer Science Institute of Singapore, Singapore 117599, Singapore; 2 Department of Haematology-Oncology, National University Cancer Institute, Singapore, National University Health System, Singapore 119228, Singapore

**Keywords:** Breast cancer, wild-type p53, mutant p53, targeted therapy, MDM2

## Abstract

The transcription factor p53 is an important regulator of a multitude of cellular processes. In the presence of genotoxic stress, p53 is activated to facilitate DNA repair, cell cycle arrest, and apoptosis. In breast cancer, the tumor suppressive activities of p53 are frequently inactivated by either the overexpression of its negative regulator MDM2, or mutation which is present in 30-35% of all breast cancer cases. Notably, the frequency of p53 mutation is highly subtype dependent in breast cancers, with majority of hormone receptor-positive or luminal subtypes retaining the wild-type p53 status while hormone receptor-negative patients predominantly carry p53 mutations with gain-of-function oncogenic activities that contribute to poorer prognosis. Thus, a two-pronged strategy of targeting wild-type and mutant p53 in different subtypes of breast cancer can have clinical relevance. The development of p53-based therapies has rapidly progressed in recent years, and include unique small molecule chemical inhibitors, stapled peptides, PROTACs, as well as several genetic-based approaches using vectors and engineered antibodies. In this review, we highlight the therapeutic strategies that are in pre-clinical and clinical development to overcome p53 inactivation in both wild-type and mutant p53-bearing breast tumors, and discuss their efficacies and limitations in pre-clinical and clinical settings.

## Introduction

### Physiological functions of p53 in cancer

#### Introduction to p53

**Figure 1:**
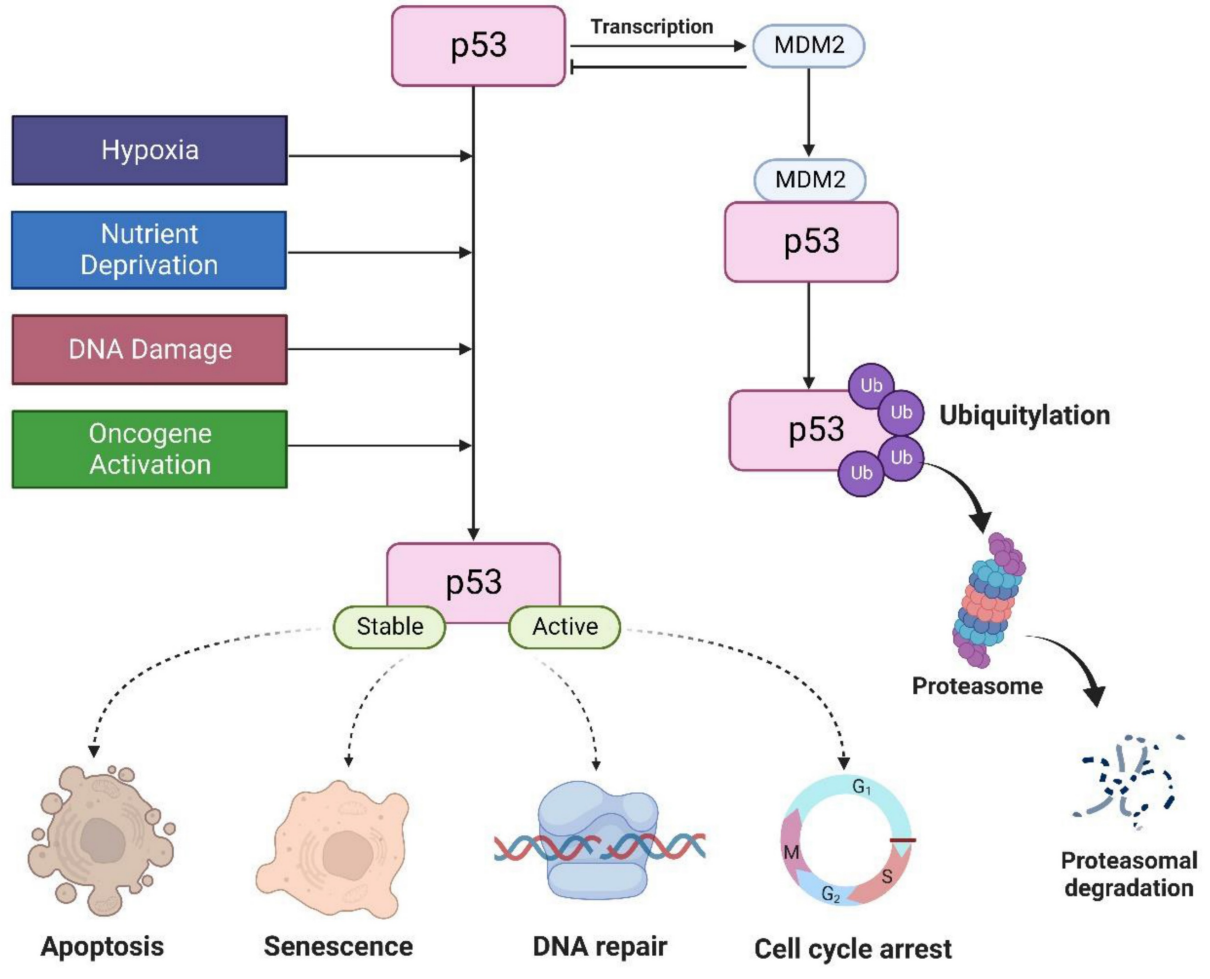
Under normal conditions, MDM2 regulates p53 protein. P53 is activated by hypoxia, activation of oncogenes, DNA damage, and nutrient deprivation to regulate cell cycle, apoptosis, DNA repair, and senescence.

The tumor suppressor p53 is a major transcription factor involved in the regulation of several cellular functions. In cancer, p53 attenuates cell proliferation in response to various stimuli, including DNA damage, nutrient deprivation, hypoxia, hyperproliferative signals, thereby preventing tumor formation 1. Thus, it is aptly known as “guardian of the genome”. Under normal physiological conditions, p53 levels are tightly regulated by MDM2, an E3 ligase that triggers p53 degradation through the ubiquitin proteasome-dependent pathway. p53 can also induce MDM2 expression by binding to its promoter. This creates a negative feedback loop to promote p53 degradation to maintain a low cellular p53 2. In the presence of cellular stresses, p53 undergoes a series of posttranslational modifications such as 1) phosphorylation which stabilizes p53 by disrupting its interaction with MDM2, 2) sequence-specific DNA binding, 3) transactivation where p53 activates or represses its target genes involved in the control of cell cycle, DNA repair, and induction of senescence and apoptosis (Figure 1) 3.

In this article, we will be looking into the involvement of p53 and its mutations in the different processes associated with breast cancer development and review the various therapeutic strategies that are being developed to target wild-type and mutant p53 cancers.

#### Functional effects of p53

*DNA repair:* As a tumor suppressor, p53 plays a vital role in maintaining genome stability by allowing time for DNA repair machinery to eliminate damaged lesions before cell proliferation resumes 4. Several DNA repair pathways regulated by p53 are the nucleotide excision repair, base excision repair, mismatch repair, and DNA double stranded break repair pathways including non-homologous end joining and homologous recombination 5.

*Cell cycle regulation:* Cell-cycle checkpoints are important to prevent cells from progressing to the next phase of cell cycle before its prior phase has been accurately completed. In the presence of DNA damage, p53 is activated to regulate the G1-S and G2-M phase restriction points. Upon activation, p53 transcriptionally regulates p21, 14-3-3σ, Cdc25c, and GADD45 to promote cell cycle arrest 6. It was reported that p53 induces G1 arrest primarily through p21 transactivation. p21 inhibits cyclin E/CDK2 function, thereby inhibiting hyperphosphorylation of retinoblastoma (Rb). Thus, Rb remains bound to E2F and the E2F-mediated transcription of cell cycle genes essential for G1 to S phase progression is inhibited. p53 also blocks G2/M by inhibiting cyclin B1/cdc2 functions through promoting 14-3-3σ expression upon DNA damage 7.

*Apoptosis:* Multiple studies have reported that p53 plays an important role in triggering apoptosis 8, 9. Both major signaling pathways that propagate apoptosis: extrinsic pathways and intrinsic pathways are mediated by p53 10. p53 triggers the extrinsic apoptotic pathway by inducing the expression of the cell death receptors Fas, Death Receptor 5, and death-domain containing receptor for TRAIL. This results in the formation of the Death-inducing signaling complex to trigger a cascade of caspase activation, including caspase-8 and caspase-3 11. p53 also triggers the intrinsic apoptotic pathway by transactivating Bcl2 family proapoptotic genes, including *BAX*, *Noxa*, *PUMA*, and *BID*. The interaction of these genes promotes multimerization and functional activation of BAX to trigger cytochrome c release, leading to the activation of caspase 9 12.

*Senescence:* Senescence is an irreversible cell cycle arrest that can be induced by different types of cellular stresses. Upon induction of senescence, p53 is activated by either ATM/ATR or Chk1/2 protein to transactivate p21, thereby activating the Rb pathway 13. However, it is worth noting that p53 levels, kinetics, and threshold of stressor intensity can dictate if a cell decides to undergo senescence or apoptosis 14.

### Role of p53 in breast cancer

#### TP53 status and prognosis in breast cancer

**Figure 2:**
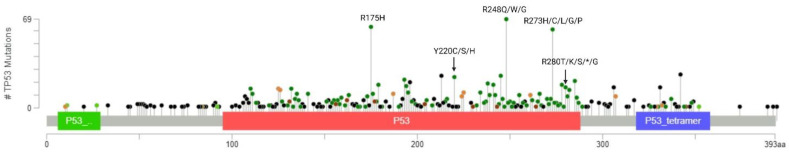
Distribution and frequency profile of *TP53* mutations in breast cancer plotted using cBioPortal. Data were pooled from TCGA and METABRIC datasets (n=3593). Mutation types and their corresponding color codes are as follows: missense mutations (green), truncating mutations (black), in-frame mutations (red), and splice variants (orange). The top 3 most frequently altered residues are R248, R175, and R273.

**Figure 3:**
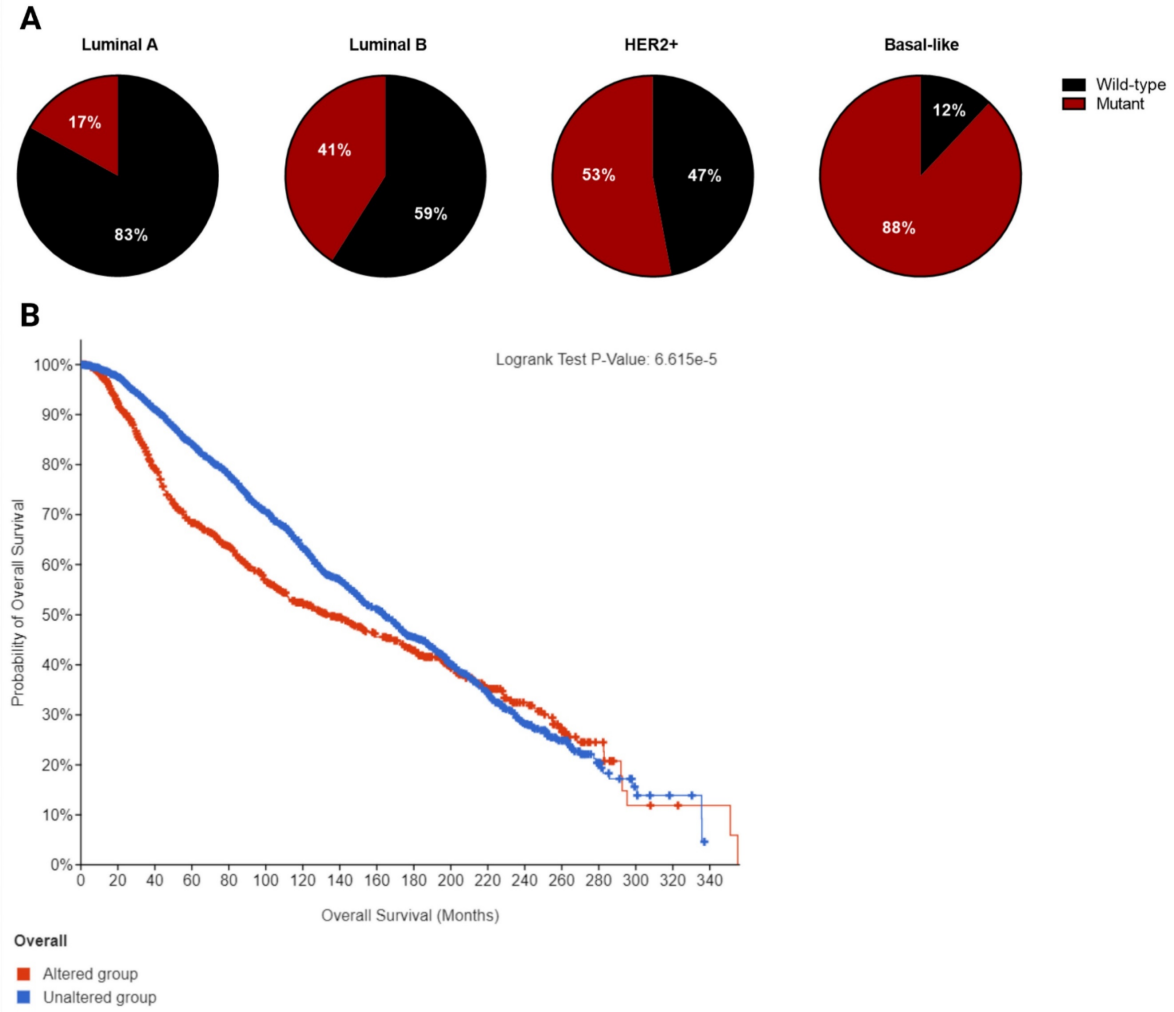
A. Frequency of p53 mutation in different breast cancer subtypes. B. Overall survival curve of altered (mutant p53, n=1018) versus unaltered (wild-type p53, n=2047) among breast cancer patients plotted using cBioPortal. Data were pooled from TCGA and METABRIC datasets (Total n= 3065). Median overall survival of patients with mutant p53 and wild-type p53 is 133.23 (95% confidence interval: 111.97 - 159.07) and 164.03 (95% confidence interval: 152.07 - 173.03) in months respectively (p=0.000114).

Breast cancer is a heterogeneous disorder composed of several biological subtypes that have well-defined characteristics and responses to therapy 15. *TP53* is the most frequently mutated gene in breast cancer, constituting approximately 30% of all breast cancer cases 16. The majority of p53 mutations are located within the DNA binding domain and are caused by missense point mutations that inhibit its transcriptional activity (Figure 2). These mutations are highly linked to tumor subtypes 17 (Figure 3A) and are associated with poor survival in breast cancer patients (Figure 3B) 18.

Breast cancers are mainly divided into four molecular subtypes based on gene expression patterns: luminal-like (luminal-A or luminal-B), basal-like, normal-like, and HER-2 positive 19. The frequency of p53 mutation is lowest in the luminal-like subtype (26%) with luminal-A having a lower mutation frequency (17%) than luminal-B (41%). In contrast, basal-like tumors have the highest frequency of p53 mutation (88%), while about half of HER2-amplified tumors (53%) harbor p53 mutations. The presence of p53 mutation and HER2 overexpression is associated with significantly worse prognosis compared to other subtypes 20.

It has also been shown that inflammatory breast cancer (50%) has a higher frequency of p53 mutations than non-inflammatory breast cancer (20-30%) 21. A recent paper reported that p53 mutations are associated with shorter overall survival in metastatic breast cancer across subtypes 22. The frequency of p53 mutations is also found to be higher in patients in advanced stage and high-grade tumors. Moreover, these mutations have been associated with subtypes with aggressive behavior such as triple negative breast cancer (TNBC) or HER2-amplified BC 23.

### Role of mutant p53 in cancer development and progression

**Figure 4:**
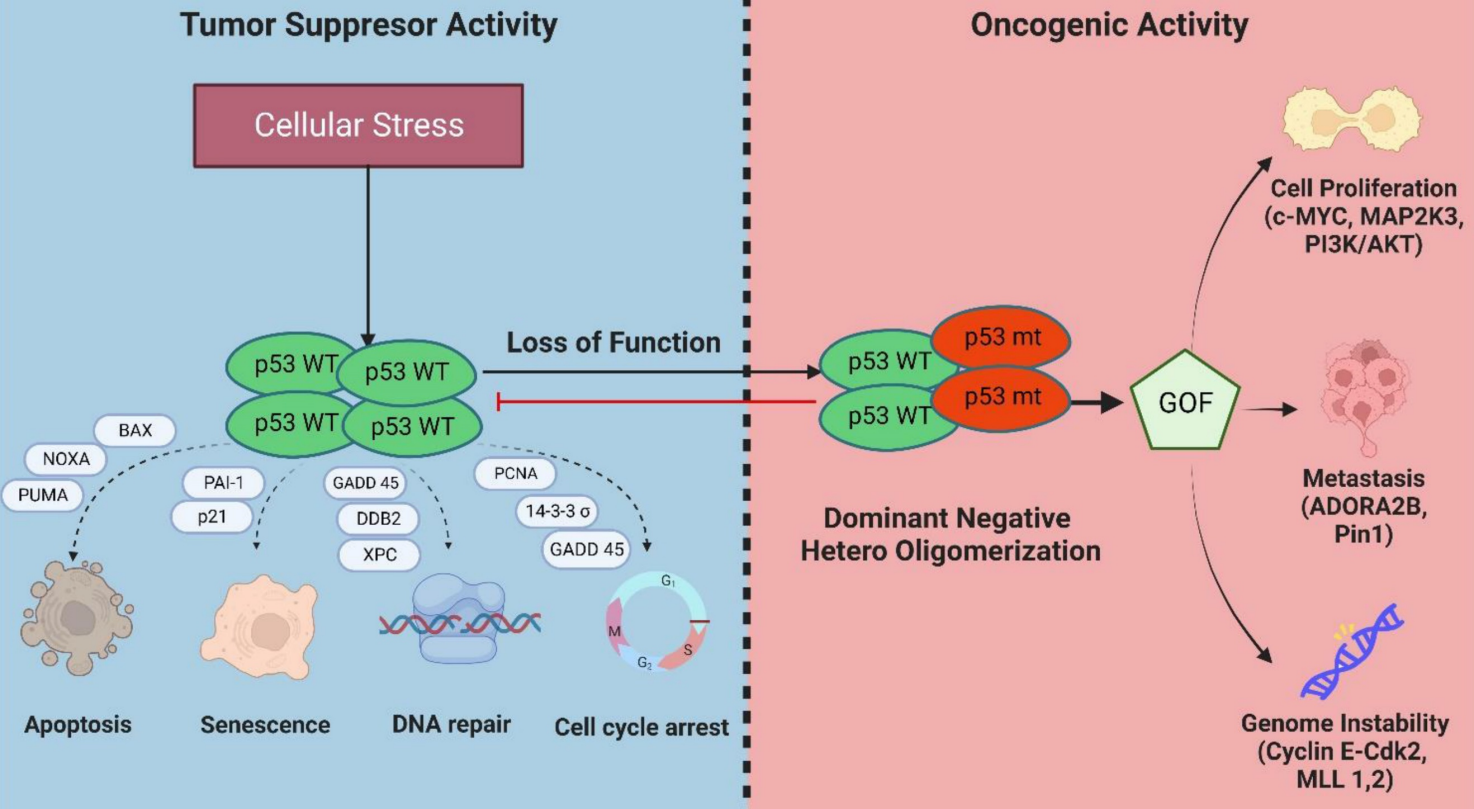
Mechanisms of inactivation of the tumor suppressive activity of wild-type p53 and GOF activities in mutant p53-bearing breast tumors.

Besides exhibiting a dominant-negative activity that obstructs wild-type p53 functions, mutations in p53 can also acquire oncogenic activity by gain-of-function (GOF) mechanisms in which the mutant protein interact with novel transcription factors or cofactors to regulate gene transcription and expression to drive cancer development (Figure 4) 24.

*Genome instability:* Genome instability is an inherent hallmark of all types of human cancers. The GOF activity of mutant p53 has been shown to promote genome instability. In breast cancer samples, mutant p53 is associated with abnormal copy numbers 25. The p53 R172H mutant (R175H equivalent in mice) shows centrosome abnormalities by modulating cyclinE-cdk2 activity at the centrosome, leading to centrosome amplification 26. Another study reported that mutant p53 can bind and increase the expression of chromatin-regulated genes, including methyltransferases MLL1 and MLL2 which enhance histone methylation and acetylation and contributes to genomic instability and cancer progression 27.

*Evading apoptosis:* Mutant p53 evades apoptosis by interacting with p53 family members, p63 and p73, to suppress their tumor suppressive activities 28. Several p53 mutations, such as the R273H and R175H, were reported to inhibit apoptosis by suppressing the expression of BCL2-modifying factor via enhanced AKT signaling 29 and reducing p73 transcriptional activity on Bax promoter respectively 30.

*Proliferation:* Mutant p53 proteins can activate the transcription of several genes associated with cell proliferation that are distinct from its wild-type counterpart 31. Several of these include *c-MYC*, *MAP2K3*, *CXCL1*, and *CCNE2*. Moreover, mutant p53 has been reported to interact with several proteins or transcription factors that drive breast cancer proliferation such as the sterol regulatory element-binding protein (SREBPs) via activation of mevalonate pathway 32, PARP 33, NF-Y 34, and DAB2IP 35.

*Metastasis:* Mutant p53 has also been reported to enhance tumor aggressiveness and metastatic potential via several mechanisms. One such mechanism is the stabilization of mutant p53 protein by Rab coupling protein-mediated secretion of Hsp90 which enhances cell invasion and metastasis in cancer cells 36. Mutant p53 also interacts with the G-protein coupled receptor, adenosine A2b receptor (ADORA2B) 37, and Pin1 38. Both interactions result in increased invasion and metastasis and are associated with poor clinical outcomes in breast cancer.

### P53 as a therapeutic target: a two-sided approach

While *TP53* is the most frequently altered gene in breast cancer, more than two-thirds of breast cancer cases retain the wild-type status, of which the majority belongs to the luminal-like subtype 16. The p53 activity in these tumors is mainly suppressed by the overexpression of MDM2 or MDMX. For the other one-third of breast cancer cases, p53 function is inactivated by mutations, of which majority are observed in HER2+ and basal-like subtypes. As such, several mutation status-differentiated therapeutic strategies have been explored to target these two main mechanisms of p53 inactivation in order to restore its tumor suppressive functions.

### Therapies against wild-type p53-bearing breast cancer

**Figure 5:**
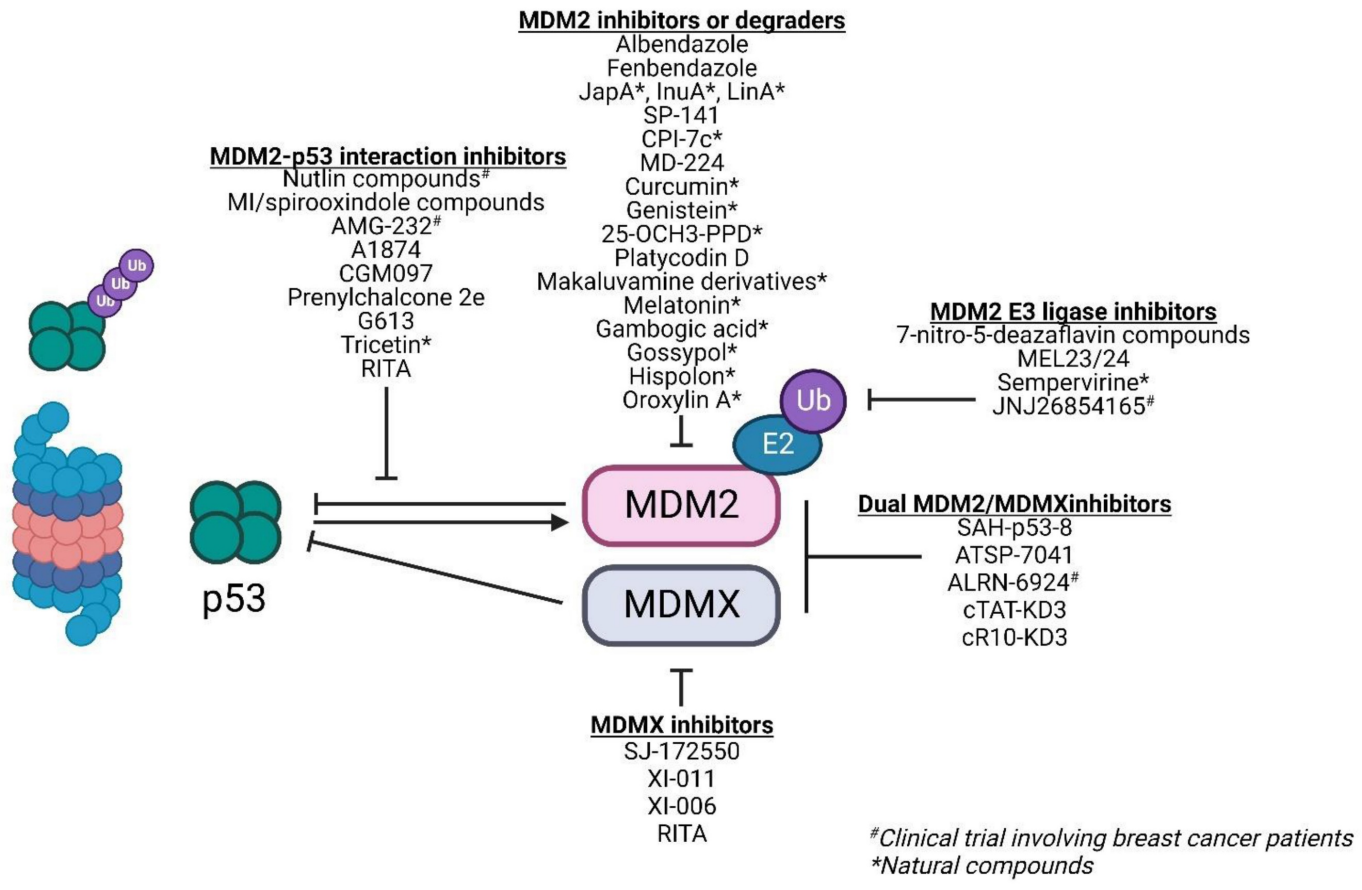
Compounds against wild-type p53 tumors that have been evaluated in breast cancer

A deregulated balance between p53 and MDM2 can lead to malignant changes in normal cells. Approximately 7% of all human tumors have *MDM2* gene amplification 39. This gives MDM2 an oncogene-like property. In breast cancer, approximately 38% of patients exhibit increased MDM2 protein expression 40. Furthermore, MDM2 expression is associated with the presence of estrogen receptors (ER) with higher MDM2 expression observed in ER+ cell lines 41. This makes MDM2 an attractive target for therapies especially in cancers with wild-type p53. Majority of therapies against MDM2 focus on either 1) inhibiting the MDM2-p53 interaction, 2) downregulating MDM2 expression, and 3) inhibiting its E3 ligase activity and its interaction with the proteasome to reduce p53 degradation and activate p53-dependent tumor suppressor pathways (Figure 5; Table 1).

#### Inhibiting the MDM2-p53 interaction

High-resolution crystal structure of MDM2-p53 binding revealed a hydrophobic surface pocket (Phe19, Trp23, Leu26) 42. This allows the development of small molecules to block the MDM2-p53 interaction, thereby activating p53 functions in cancer cells. There are over 20 different chemical classes that claim to inhibit such interaction 43. However, most studies focus on three classes of compounds: nutlins, spirooxindoles, and benzodiazepinediones. In this review, we will focus our discussions on nutlin-based and spirooxindole class compounds given that they are the most widely used classes among the MDM2-p53 interaction inhibitors in breast cancer studies and display drug-like properties.

*Nutlins:* Nutlins act by sterically inhibiting MDM2 at its p53 binding pocket. This in turn stabilizes p53 and induces p53-associated cell cycle arrest and apoptosis in MDM2-overexpressing cancer cells 44. Nutlin-1, 2, 3 are the first of such compounds of the nutlin family. Over time, there have been multiple iterations of the nutlin compound including RG7112 and Idasanutlin (RG7388) with improved binding, potency, and pharmacological properties 45. While these compounds showed dose-dependent stabilization of p53 and sensitivity in cells with wild-type p53, they failed to induce any p53-regulated tumor suppressive effects in cells harboring mutant or non-functional p53 46.

The activity of nutlins is limited by the overexpression of MDMX, another p53 negative regulator, as they exclusively bind to MDM2's p53 binding pocket 47. A strategy to overcome p53 inactivation by MDMX is combining nutlins with doxorubicin, an anthracycline-based DNA damaging agent that triggers direct MDM2 ubiquitination and subsequent degradation of MDMX 48. Yet another strategy of overcoming MDMX inactivation is by using small molecule MDMX inhibitors such as SJ-172550, XI-011, and XI-006. These inhibitors yield additive effects in combination with nutlin-3a and induce apoptosis in the ER+ MCF-7 cell line with wild-type p53 49, 50.

Several nutlin-based compounds have advanced into Phase I and Phase II clinical trials, however, most of these are evaluated in patients with hematological malignancies or sarcomas. To our knowledge, there is only one trial recruiting exclusively breast cancer patients (NCT03566485). This study investigated the combination of idasanutlin (100mg oral) and anti-PD-L1 immune checkpoint inhibitor atezolizumab (840mg IV) in stage IV or unresectable recurrent ER+ breast cancer patients. The rationale behind this drug combination stems from the observation that MDM2 amplification is associated with rapid disease progression in patients receiving PD-1/PD-L1 inhibitors in various cancers 51. Unfortunately, this study has been terminated due to low accrual. Preliminary results showed 4 out of 7 patients with disease progression and serious adverse events in which the majority are associated with blood or lymphatic disorders, suggesting that there are still major hurdles to overcome before nutlin-based compounds can be applied in the clinic for breast cancer patients.

*Spirooxindoles:* Similar structural derivatives of nutlins are the spirooxindoles (or MI-series). These mimic the MDM2-p53 hydrophobic pocket residues important for binding. Similar to nutlins, drugs from this class exhibit specific potency in cells with wild-type p53 while minimally affecting those with mutant p53. There are several of such small molecule compounds evaluated in breast cancer models, however, all are in the pre-clinical stages 52-59. These are summarized in Table 1. All of these compounds exhibited anti-proliferative effects exclusively in wild-type p53-harboring breast cancer cell lines or xenografts by triggering p53 downstream genes involved in cell cycle arrest and apoptosis.

*Other small molecule MDM2-p53 inhibitors:* Besides nutlins and spirooxindoles, there are also several small molecule MDM2-p53 inhibitors that have been evaluated in breast cancer models. However, these have not been extensively studied as a class. One such example is RITA (reactivation of p53 and induction of tumor cell apoptosis) which binds to the N-terminus of p53 and induces a conformational change that disrupts the MDM2-p53 interaction, resulting in p53 accumulation 60. RITA has also been reported to induce MDMX degradation through ATM 61. Other compounds that act on the MDM2-p53 interaction include prenylchalcone 2e, tricetin, and NVP-CGM097. These compounds lead to increased protein expression of p53 and its downstream effectors, inducing cell cycle arrest and apoptosis specifically in wild-type p53 bearing cells 62-64.

The MDM2-p53 piperidinone-based inhibitor, AMG 232 (or KRT-232), was evaluated in a Phase I trial recruiting patients with wild-type p53 advanced cancers, including ER+ breast cancer (NCT01723020). These patients received AMG 232 at the maximum tolerated dose of 240 mg with 3 patients exhibiting dose-limiting toxicities of thrombocytopenia or neutropenia. AMG 232 treatment increased serum macrophage inhibitor cytokine-1, indicating p53 pathway activation and on-target activity, in a dose-dependent manner. Promising efficacy signals were observed with stable disease in 7 out of 12 ER+ breast cancer patients (58.3%) 65.

*PROTACs:* The concept of MDM2-p53 binding inhibition has extended beyond small molecules. An example is the use of these compounds for the design of MDM2-based proteolysis targeting chimeras (PROTACs), utilizing MDM2's E3 ligase activity to degrade a specific protein-of-interest. There are currently 3 PROTACs which incorporate a nutlin-based derivative to recruit MDM2 for the degradation of androgen receptor, PARP1, and BRD4 respectively 66. Besides degradation of pro-oncogenic proteins, these compounds have a dual role in sequestering MDM2, thereby stabilizing wild-type p53 in the process. PROTACs can also be designed to target MDM2 for degradation. The IMiD-based PROTAC MD-224 utilizes lenalidomide and MI-1061 as ligands of the E3 ligase cereblon and MDM2 respectively to degrade MDM2 protein which in turn decreases p53 degradation by MDM2 67.

*Peptides:* Other non-small molecule MDM2-p53 inhibitors include angler peptides developed by Phillippe and colleagues. Angler peptides were synthesized by conjugating cell-penetrating peptides with the cell-impermeable peptide inhibitor of MDM2-p53/MDMX-p53, KD3. Two of such peptides, cTAT-KD3 and cR10-KD3, were able to activate the p53 pathway via endocytic pathway and direct membrane translocation within micromolar concentrations respectively in several cancer cell lines including MCF-7. This study showed that non-permeable peptides can be conjugated to a stable penetrating peptide to allow delivery into cytosol to inhibit specific pathways such as MDM2/MDMX-p53 pathway 68.

Stapled peptides can also be utilized to inhibit the MDM2-p53 interaction. The main selling point of stapled peptides is that they can inhibit both MDM2 and MDMX. Examples of stapled peptides that have been evaluated in breast cancer models include SAH-p53-8 which exhibited better response in MCF-7 compared to Nutlin-3 69 and ATSP-7041 which was reported to induce a prolonged inhibitory effect of MDM2-p53 interaction in MCF-7 with favorable pharmacokinetic properties 70. ALRN-6924, an analogue of ATSP-7041, was also evaluated in breast cancer cell lines and xenografts. Unsurprisingly, this peptide was selectively active in wild-type p53 and not mutant p53 cells in the nanomolar range. ALRN-6924 is the only non-small molecule MDM2-p53 inhibitor that has been evaluated in clinical trials involving breast cancer patients with wild-type p53 (NCT03725436), however, no results have been posted thus far.

#### Downregulating MDM2 expression

The inhibition or downregulation of MDM2 is another therapeutic strategy against tumors with wild-type p53. While this strategy yields a similar outcome of increased p53 activity, the mechanism of action differs from inhibition of MDM2-p53 interaction such that it directly inhibits MDM2 43.

Several compounds capable of direct MDM2 binding to induce its downregulation or degradation include JapA 71, Inulanolide A, and lineariifolianoid A. These compounds not only induce MDM2 protein degradation and represses its transcription, the latter two also inhibit NFAT1, an MDM2 transactivator frequently overexpressed in breast cancer 72, 73. Another example is SP-141 which shows direct MDM2 binding and inhibition of its expression by promoting its autoubiquitination and degradation 74. These compounds induce cytotoxicity, apoptosis, and G2/M arrest, as well as inhibit tumor growth in xenograft models irrespective of p53 mutational status. Another MDM2 inhibitor CPI-7c was reported to bind to the RING and N-terminal domains of MDM2 to induce its degradation via the ubiquitin-proteasome pathway. This not only stabilizes p53 but promotes the binding of p53 to the promoters of death receptors, DR4 and DR5, to induce TRAIL-induced apoptosis 75.

Beyond novel MDM2-targeting compounds, several existing clinically approved drugs also exhibit secondary activities against MDM2, such as albendazole and fenbendazole, which upregulate p53 and its downstream effector, p21, by significantly decreasing the expression of MDM2 and MDMX in melanoma and breast cancer cell lines 76. The widely-used selective estrogen receptor degrader, fulvestrant, was also reported to cause a decreased estrogen-dependent MDM2 upregulation and increased MDM2 protein turnover rate without altering *MDM2* gene expression and p53 activity. The reduction of MDM2 by fulvestrant is independent of p53 mutational status 77. This further strengthens its use as an effective treatment in advanced ER+ breast cancers where MDM2 is often overexpressed.

Natural compounds also exhibit activities against MDM2. Natural products such as curcumin 78, genistein 79, ginsenosides (25-OCH3-PPD 80), Platycodin D 81, makaluvamine analogues (FBA-TPQ, PEA-TPQ, MPA-TPQ, DPA-TPQ, BA-TPQ) 82, 83, melatonin 84, gambogic acid 85, gossypol 86, 87, hispolon 88, and oroxylin A 89 have all been shown to downregulate MDM2 expression; however, their association with MDM2 were all only evaluated in the pre-clinical stages.

While direct targeting of MDM2 is a viable therapeutic strategy against wild-type p53 tumors, the approaches are still in the pre-clinical stages. It is worth highlighting that both wild-type and mutant p53-carrying cells respond to MDM2 inhibition across the studies mentioned in this review. A proposed mechanism of action is p21 upregulation independent of p53. As such, the use of direct MDM2 inhibitors may provide an edge over inhibitors of the MDM2-p53 interaction in mutant p53 tumors.

#### Inhibiting MDM2's E3 ligase activity and its binding to proteasome

Another strategy to minimize the degradation of wild-type p53 is through inhibition of E3 ligase activity of MDM2 or its binding to the 26S proteasome. The family of 7-nitro-5-deazaflavin compounds (HLI98s, HLI373, and HLI393) promotes p53 stabilization by binding to MDM2's RING finger domain that is responsible for its E3 ligase activity without depleting its expression. This results in the inhibition of MDM2-mediated p53 degradation, restoring p53 activities to selectively kill cancer cells harboring wild-type p53 90, 91. Similarly, the natural compound sempervirine inhibits MDM2 E3 ligase activity resulting in apoptosis of cancer cells 92. Another class of MDM2 E3 ligase inhibitors are the MEL compounds MEL23 & MEL24 which inhibit MDM2-MDMX E3 ligase activity by decreasing the ubiquitination of the RING domains of MDM2-MDMX heterocomplex. While they induced cell death in a p53-dependent manner in cells harboring wild-type p53, they also exhibited p53-independent/MDM2-dependent activity in p53-null cells, suggesting that these compounds can be used beyond wild-type p53-expressing cancers 93.

The tryptamine derivative, JNJ26854165, functions to inhibit the binding of MDM2-p53 complex to proteasome, thereby inducing p53-associated apoptosis in cells with wild-type p53 93. This compound has successfully advanced into a Phase I trial including seven patients with advanced breast cancer (NCT00676910). One patient showed partial response to JNJ26854165, and tumor biopsies showed 102% increase in p53 levels 94. JNJ26854165 was also reported to exhibit other anti-tumoral activities beyond MDM2/p53 as it induced S-phase arrest and E2F1-mediated apoptosis in cells with p53 mutation(s) 95.

### Therapies against mutant p53-bearing breast cancer

Mutant p53 drives tumor growth, progression, and metastasis, and is frequently found in HER2-enriched and TNBC subtypes 96. This makes it an attractive therapeutic target for breast cancer. Mutation of p53 is also likely clonal in TNBC 97, providing a rare feature of homogeneity in a heterogenic cancer like breast cancer. The relative lack of targeted therapies and intrinsic aggressiveness of TNBC tumors further underscore the therapeutic value to target mutant p53.

**Figure 6:**
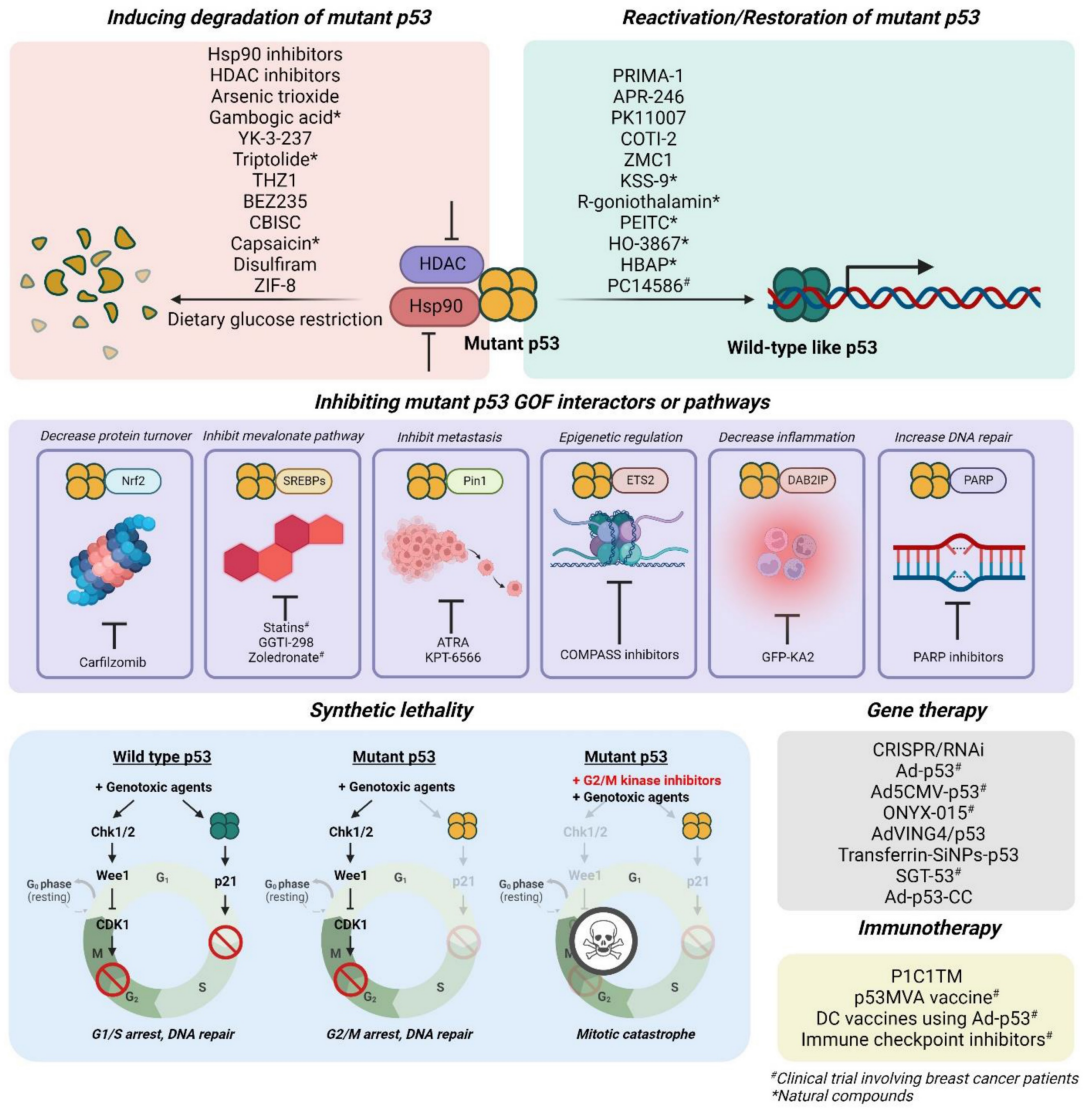
Strategies to treat mutant p53 tumors that have been evaluated in breast cancer

Targeted therapies against tumors with mutant p53 mainly focus on 1) reactivation or restoration of mutant p53 to wild-type p53 functions, 2) promoting degradation, 3) inducing synthetic lethality, 4) inhibiting its gain-of-function-associated interactors or pathways, 5) immunotherapies, and 6) gene therapies with vectors to incorporate wild-type p53 gene into tumor cells (Figure 6; Table 2).

#### Restoring mutant p53 to wild-type-like conformations or functions

Majority of *TP53* mutations are missense with a single amino acid substitution in the p53 protein, leading to a loss of its DNA binding ability 98. Various compounds such as quinuclidines, pyrazoles, 2-sulfonylpyrimidines, and thiosemicarbazones have been developed to restore mutant p53 to wild-type p53-like functions.

*Quinuclidines:* Quinuclidine-based prodrugs, PRIMA-1 and its methylated analogue, APR-246 (or PRIMA-1^MET^), are metabolized* in vivo* into the active metabolite, 2-methylene-3-quinuclidinone (MQ). MQ is a nucleophile acceptor that modifies thiol groups via Michael addition reaction, correcting the folding of mutant p53 protein by targeting exposed cysteine residues within its core domain to restore its DNA binding capability. Known targets of MQ include Cys-124 and Cys-277 which are key residues in stabilizing mutant p53 R175H 99. Besides mutant p53 reactivation, PRIMA-1 also reverses its dominant negative effect by preventing the aggregation of mutant p53 100. Additionally, it upregulates p53 downstream genes such as *p21*, *BAX*, *PUMA*, and *NOXA*, and activates caspase-2, -3, and -9 to induce apoptosis in several cancers including breast cancer 101.

Both PRIMA-1 and APR-246 were reported to preferentially inhibit mutant p53 over wild-type p53 breast cancer cell lines and mice xenografts 100. Interestingly, high endogenous p53 expression also correlates with increased sensitivity to APR-246 in a range of breast cancer cell lines 102.

Currently, PRIMA-1 and its analogue are a subject of future studies as not all targeted cysteine residues within the core domain have been identified and their mechanisms of reactivating mutant p53 remain unclear. Furthermore, the modification of thiol groups by MQ are not specific. MQ can also react with thioredoxin reductase 1 and deplete glutathione, generating reactive oxygen species (ROS) and induce cytotoxicity independent of p53 mutation 103.

*2-sulfonylpyrimidines:* The 2-sulfonypyrimidine compound, PK11007, also exhibits mutant p53 restoration properties via selective alkylation of cysteine residues, which increases thermal stability of DNA binding domain of mutant p53 Y220C and V143A 104. PK11007 was also reported to preferentially induce apoptosis and exhibit growth inhibitory effects in mutant p53 over wild-type p53 carrying breast cancer cells at a significantly lower concentration. RNA sequencing and gene ontology data revealed that PK11007 increased gene expression in pathways associated with apoptosis, signal transduction, protein refolding, and locomotion, induced by p53 reactivation 105.

*Thiosemicarbazones:* Another class of compounds that reactivates mutant p53 are the thiosemicarbazones. Zinc ion is an essential cofactor of wild-type p53 to facilitate p53 binding to DNA. Mutations in any of the zinc-coordinating residues abolish p53 binding to zinc, resulting its inability to bind to DNA 106. Thiosemicarbazones act as zinc chelators, promoting zinc binding to mutant p53. COTI-2 is one such compound of this class. It has been shown to restore DNA binding capacity of mutant p53, thereby inducing activation of p53 target genes including *p21*, *PUMA*, and *NOXA*. *In vitro* data revealed that breast cancer cell lines harboring mutant p53 exhibit significantly lower IC_50_ than those with wild-type p53 107. COTI-2 was reported to be safe and well-tolerated in various mice xenografts, including MDA-MB-231 mutant p53 model. Other thiosemicarbazone-derived compounds including NSC319725, NSC319726, NSC328784. NSC319726 (or ZMC1) preferentially inhibited p53 mutant cell lines over wild-type cells, specifically those with the R175 mutation 108. Like APR-246 and PK11007, NSC319726 was reported to decrease glutathione levels which in turn increases ROS.

*Natural p53 reactivators:* There are several naturally occurring compounds that show mutant p53 restoration activities. These include KSS-9 109, R-goniothalamin (GON) 110, Phenethyl Isothiocyanate (PEITC) 111, HO-3867 112, chetomin 113, and 2-[(4-hydroxybenzyl) amino] phenol (HBAP) 114. These compounds are capable of restoring the transcriptional activity of p53 and exhibit preferential activities towards mutant p53 over wild-type p53 harboring cell lines in various models of breast cancer under *in vitro* and/or *in vivo* conditions.

Several of these small molecule reactivators of mutant p53 have advanced to Phase I and II trials, including APR-246, COTI-2 and PC14586. However, only PC14586 has been validated in breast cancer patients. The oral, small molecule inhibitor, PC14586, was administered in patients with advanced solid tumors with p53 Y220C mutation, including several breast cancer patients, as part of PMV Pharmaceuticals' Phase I/II clinical trial (NCT04585750). This compound is designed to specifically target Y220C by increasing its thermal stability, thus restoring p53 DNA binding abilities. Preclinical results showed a restoration of p53 transcriptional activity and increased expression of *p21*, *MDM2*, *Bax*, and *PUMA*, and oral administration of PC14586 at 100mg/kg in nude mice bearing Y220C gastric cancer xenografts resulted in 80% tumor regression after 3-weeks 115. Initial Phase I data concluded that the compound is generally safe and well-tolerated up to 3000 mg daily with one of the breast cancer patients achieving partial response 116, showing promise of this therapeutic strategy.

#### Inducing mutant p53 degradation

*Hsp90 inhibitors:* Unlike normal cells, the stabilization of mutant p53 proteins in cancer cells are aided by chaperones such as heat shock protein 90 (Hsp90) 117. Thus, targeting Hsp90 may be a strategy against mutant p53 tumors. The first developed inhibitors of Hsp90, geldanamycin and its analogue 17-AAG, were reported to destabilize p53 V143A, R175H, S241F, R273C/H, and R280K mutations, thereby triggering its degradation by E3 ligases MDM2 or CHIP 118. Ganetespib was later synthesized with much improved potency and was reported to induce mutant p53 degradation in breast cancer mice models encompassing several p53 mutations 119.

*HDAC inhibitors:* Inhibitors of histone deacetylase (HDAC), besides their role in epigenetic regulation, were reported to exhibit secondary activities against mutant p53 via transcriptional and post-transcriptional mechanisms. Vorinostat (SAHA) and NaB are examples of Class I HDAC inhibitors that block the transcription of mutant p53 by abolishing the activity of HDAC8 and Yin Yang-1 complex in TNBC 120. Additionally, vorinostat induces p53 degradation by reducing its stability via inhibition of HDAC6, a key regulator of Hsp90 119. Other HDAC inhibitors such as Trichostatin A, sodium butyrate, and FR901228 were reported to reduce protein levels of p53 R175H, P223L, V274F, and R249S, and R280K mutants 121.

While Hsp90 and HDAC inhibitors have been evaluated in various clinical trials involving breast cancer patients, their association with p53 mutational status has not been fully investigated.

*Other inducers of mutant p53 degradation:* Besides Hsp90 and HDAC inhibitors, there are other natural or synthetic compounds that show mutant p53 degradation activities in various breast cancer models. These include arsenic trioxide 122, gambogic acid 123, Spautin-1 124, YK-3-237 125, triptolide, *Origanum majorana* extract (OSE) 126, 127, THZ1 128, BEZ235 129, CBISC 130, capsaicin 131, and disulfiram 132. However, for most of these compounds, the mechanisms are not yet known or they exhibit broad effects that may not be specific to mutant p53.

There are also other means of inducing mutant p53 degradation beyond the use of small molecule compounds. For example, zeolitic imidazolate framework-8 (ZIF-8), composed of zinc ions as the metal node and 2-methylimidazolate as its linker, was reported to increase mutant p53 gluthionylation to facilitate polyubiquitination and its subsequent degradation. ZIF-8 was later improved using Z1-RGD peptide to exhibit higher stability and better internalization, as well as mutant p53 degradation ability. This modified ZIF-8 demonstrated promising therapeutic efficacy in breast cancer patient-derived xenograft (PDX) carrying p53 mutation 133. Another means of degradation is through dietary glucose restriction. Mutant p53 drives the Warburg effect by promoting the translocation of glucose transporter GLUT1 to the cell surface to increase glucose uptake. Glucose restriction can trigger mutant p53 deacetylation and degradation, thus preventing its accumulation which in turn slows down tumor growth in mice models 134.

#### Synthetic lethality

The G1/S checkpoint is an important cell cycle regulator to ensure proper DNA replication 135. In the presence of DNA damage, wild-type p53 will induce G1/S arrest to repair DNA before S phase entry 5. Cancer cells harboring mutant p53 can bypass this checkpoint even with DNA damage. Thus, they rely entirely on the G2/M checkpoint to maintain genomic stability 136. An inactivation of G2/M checkpoint will result in unscheduled entry into mitosis even with extensive DNA damage, causing mitotic catastrophe. This can be a potential therapeutic target for mutant p53 tumors.

*Targeting the G2 checkpoint:* Wee1 kinase plays a critical role in G2/M checkpoint control by mediating cell cycle arrest through inhibiting CDK1 activity. Wee1 is upregulated in various cancers including breast cancer 137. The Wee1 inhibitor, MK-1775 (or AZD1775 or Adavosertib), promotes cancer cells to bypass G2/M checkpoint, leading to mitotic catastrophe. This compound showed amplified anti-tumor activity in cells harboring mutant p53 over wild-type p53 in various cancer models, however, this difference is not as notable amongst ER+ cell lines 138, 139.

Another compound that exploits mutant p53's reliance on G2/M is UCN-01, a Chk1 (upstream of Wee1). While this compound showed preferential cytotoxic effect in mutant p53-overexpressing cells to wild-type p53, it yielded less than satisfactory results in Phase I clinical trials 140. A more selective inhibitor of Chk1, AZD7762, revived Chk1 as a therapeutic target against mutant p53 as it exhibited enhanced response and increased survival in TNBC mice models bearing p53 mutation 141. Additionally, AZD7762 promotes radiation-induced apoptosis and mitotic catastrophe in mutant p53 T47D breast model, while impeding tumor xenograft growth by abrogating radiation-induced G2/M arrest and DNA double-strand repair 142.

The G2 checkpoint vulnerability of mutant p53 also forms the basis of several combination therapy designs in breast cancer. For example, the sequential treatment of a pan-CDK inhibitor, roscovitine before doxorubicin was shown to induce synthetic lethality specifically in mutant p53 TNBC cells. Roscovitine arrests these cells in G2/M phase, and in combination with doxorubicin, increases DNA double-strand breaks and inhibits DNA repair by decreasing homologous recombination-associated proteins. The sequential treatment of roscovitine and doxorubicin shows superior reduction in tumor volume and improved overall survival in mutant p53 TNBC xenografts over single-agent or concomitant treatment 143. Another combination therapy using deoxyuridine analogues and PARP inhibitors also enhances DNA damage. However, unlike wild-type p53 cells, cells with mutant p53 are unable to activate the p53-p21 signaling pathway to arrest at G1 for DNA repair. As a result, these cells are arrested at G2 with accumulation of DNA damage. This combination is unsurprisingly synergistic only in mutant p53 breast cancer and significantly inhibits growth and improves overall survival in mutant p53 TNBC xenografts 144.

*Other targets of synthetic lethality:* The concept of synthetic lethality in mutant p53 extends beyond G2 dependence. Computational gene expression profiling revealed multiple candidate gene targets involved in synthetic lethality with mutant p53. Among these candidate genes are *PLK1*, *PLK4*, and *AURKA* of which inhibitors are available 145. Indeed, the treatment with PLK1 inhibitor Onvansertib showed a more profound effect in mutant p53-prevalent TNBC compared to wild-type p53-prevalent luminal cell lines. Onvansertib in combination with paclitaxel is also selectively synergistic in mutant p53 breast cancer cell lines and xenograft 146. Another synthetic lethal target is phosphatidylinositol 5-phosphate 4-kinase (PI5P4K). PI5P4K was reported to be amplified in a subset of breast cancers and is associated with p53 mutations or deletions. Depletion of this kinase in p53-deficient breast cancer cells resulted in growth inhibition *in vitro* and prolonged lifespan of p53-knockout mice 147. So far, THZ-P1-2 is the only available inhibitor of PI5P4K, however, it has not been studied in breast cancer. Besides the mentioned targets, there may be other synthetic lethal targets associated with mutant p53 remain to be discovered. These discoveries may provide more therapeutic options against tumors with mutant p53.

#### Inhibiting mutant p53 gain-of-function interactors or pathways

With the knowledge of mutant p53 interactors, another therapeutic strategy is to design inhibitors of pathways or interactions associated with the GOF activities of mutant p53.

*Mutant p53-Nrf2:* The mutant p53-Nrf2 complex drives the transcriptional activation of proteasome subunit genes, thereby increasing protein turnover in breast cancer models 148. The use of proteasome inhibitor carfilozomib in combination with APR-246 was reported to reduce primary tumor growth and dissemination in mutant p53-bearing breast cancer xenografts 149.

*Mutant p53-SREBPs:* The SREBP-mutant p53 complex activates SREBP target genes, leading to increased sterol production via the mevalonate pathway that activates YAP/TAZ transcription cofactors to promote cell proliferation 32. The use of cholesterol-lowering drugs such as statins was reported to induce cytoplasmic relocalization of YAP/TAZ in breast cancer cells harboring mutant p53 150. Additionally, they were reported to degrade mutant p53 and inhibit its GOF activities. Downstream targets of the mevalonate pathway can also be targeted against mutant p53 tumors. The use of mevalonate/RhoA axis inhibitors such as geranylgeranyl transferase 1 inhibitor, GGTI-298, and the farnesyl di-phosphate synthase inhibitor, zoledronic acid (ZA), are able to mimic the phenotypic effects of statins on mutant p53 151. To date, there have been two clinical trials exploiting the interaction between SREBP and mutant p53 (NCT03358017 and NCT02347163), evaluating ZA in TNBC patients, however, no results have been posted thus far.

*Mutant p53-Pin1:* Another protein involved in mutant p53's GOF activity in breast cancer is Pin1. The structural changes of mutant p53 induced by Pin1 shape its interactome and transcriptome. Pin1 also interacts with mutant p53 to increase expression of genes such as* DEPDC1* to drive migration and invasion of breast cancer cells 38. Pin1 inhibitors such as all-trans retinoic acid (ATRA) and KPT-6566 showed a dampening of Pin1-dependent oncogenic phenotypes and growth *in vivo*, as well as curbed self-renewal capabilities of breast cancer stem cells. The sensitivity of breast cancer cells to KPT-6566 also correlates with Pin1 expression 152.

*Mutant p53-PARP:* PARP1 interacts with mutant p53, specifically the R273H mutant, at its C-terminal domain (CTD). This interaction takes place in replicating DNA and causes a negative regulation of DNA repair 153. The use of PARP inhibitor talazoparib synergizes with the alkylating agent, temozolomide to induce cytotoxicity in R273H-bearing MDA-MB-468 cell line. This synergistic sensitivity was enhanced upon R273H overexpression 33. Moreover, the deletion of CTD of R273H disrupts the R273H-PARP1 interaction, causing a reduced ability of cells to proceed pass the G2/M checkpoint, slower proliferation, and decreased sensitivity to the talazoparib-temolozomide combination 154. These results highlighted the clinical benefit of PARP inhibitors against breast cancers, especially those that express R273H p53 mutation.

*Mutant p53-DAB2IP:* DAB2IP is a tumor suppressor and is a negative modulator of PI3K/AKT signaling. Mutant p53 can bind DAB2IP, inhibit its activity, and induce AKT activation in hormone-independent breast cancer cells 35. The use of peptide aptamers such as the chimeric decoy protein (GFP-KA2) to target the mutant p53-DAB2IP complex has been shown to significantly reduce inflammation-driven and insulin-driven invasion *in vitro* and xenograft growth and spread in breast cancer models* in vivo* 155.

*Mutant p53-COMPASS:* Mutant p53 drives global epigenetic alterations in breast cancer. The interaction between mutant p53 and the transcription factor ETS2 increases the expression of chromatin-regulatory COMPASS (complex proteins associated with Set1) complex subunits, MLL1, MLL2, and MOZ, which possess histone methyl- and acetyltransferase activities respectively. Such histone modifications in turn drive proliferation. Knockdown of *MLL1* impairs colony formation and reduces tumor formation ability in GOF mutant p53 cells with minimal effect in wild-type p53 cells 27. The use of COMPASS inhibitors was reported to reduce cell growth associated with mutant p53 *in vitro*, however, its effect in breast cancer models have not been elucidated.

Overall, while these inhibitors show some degree of selectivity against mutant p53 models, this is an indirect approach of targeting mutant p53. Given the vast extent of mutant p53 interactors (which may be mutation-specific) and their varying expressions between patients, targeting multiple interactors may be required to yield a substantial clinical efficacy.

#### Genetic approaches

The introduction of viral or plasmid vectors to incorporate wild-type p53 gene can be directed towards tumor cells to trigger senescence or cell death 156. One of the most notable constructs is Gendicine (or Ad-p53) which combines adenovirus serotype-5 vector with human wild-type p53 gene. Gendicine in combination with the EGFR inhibitor, gefitinib, showed synergistic growth inhibition *in vitro*, along with decreased tumor burden of MDA-MB-468 xenograft 157. Gendicine has been evaluated as treatment for TNBC in a clinical trial, in which half of the patients were treated with Gendicine in combination with capecitabine, and another half with capecitabine alone. Gendicine + capecitabine induced higher response rates of 84.4% than that of capecitabine alone (71.8%), suggesting that Gendicine has clinical potential as treatment against mutant p53-prevalent TNBC 158. Other similar viral-based constructs are Advexin (or Ad5CMV-p53) and ONYX-015, both of which have also been evaluated in clinical trials. The intratumoral injection of Ad5CMV-p53 combined with docetaxel and doxorubicin in a Phase II trial was terminated early as none of the locally advanced breast cancer patients achieved a pathologic complete response. Despite that, all 12 patients achieved an objective clinical response with extensive tumor-infiltrating leukocytes in scattered tumor cells 159. A Phase I trial evaluated ONYX-015 in combination with anti-TNF-α receptor etanercept in patients with solid tumors, which includes 2 breast cancer patients. Four out of 9 patients showed stable disease with mild side effects 160.

Over the years, gene therapy has advanced to multigene-based combination such as the combination of p53 and inhibitor of growth 4 (ING4), another tumor suppressor that enhances p53 acetylation and its transcriptional activity. A multiple promoter expression cassette-based recombinant adenovirus co-expressing ING4 and p53 (AdVING4/p53) was evaluated in mutant p53 TNBC cell line and xenograft and showed growth inhibition and apoptosis via enhanced acetylated p53 activity and its downstream effectors 161.

Besides viral vectors, plasmid vectors can also be delivered into cancer cells using nanoparticles in p53 gene therapies. One example is the transferrin-SiNPs-p53, a transferrin-modified nanoparticle carrying the wild-type p53 plasmid construct. The treatment of MCF-7 with transferrin-SiNPs-p53 increases wild-type p53 expression and induces growth inhibition and apoptosis* in vitro* and *in vivo* 162. Another example is SGT-53, a non-viral nanocomplex composed of a cationic liposome coated with an anti-transferrin receptor single-chain antibody fragment, which can selectively deliver wild-type p53 cDNA into tumor cells based on the higher transferrin receptor on tumors absent in normal cells. This nanocomplex showed significant anti-tumor activity in several preclinical models 163, 164 and was reported to be well-tolerated and exhibited anti-tumor effects in several clinical trials involving patients with advanced solid tumors 165, 166.

While genetic approaches using vectors containing wild-type p53 show promising results, they are limited to p53-deficient tumors that do not harbor any GOF or dominant-negative activities of mutant p53 167. That is until the discovery of a chimeric superactive p53 (p53-CC). The p53-CC possesses an alternative oligomerization domain that bypasses the dominant negative effect of mutant p53 by forming homo-oligomers with each other. Its delivery was achieved using adenovirus (Ad-p53-CC) and was shown to induce superior cell death over wild-type p53 construct in a dominant negative p53 breast cancer model, MDA-MB-468. Additionally, tumor regression of MDA-MB-468 was observed after Ad-p53-CC administration, compared to Ad-p53-WT which only halted tumor growth 168. This presents an alternative strategy to overcome the dominant negative effect of mutant p53.

Other modes of correcting p53 mutation in cancer cells are by synthetic small interfering RNA (siRNA) and CRISPR-Cas9. A study has shown that the use of specific siRNA to different p53 hotspot mutations decreased cancer cell viability with no effect on wild-type p53 mRNA 169. Furthermore, base editing using CRISPR-Cas9 successfully corrected p53 mutation in HCC1954 breast cancer cell line at a 3.3-7.6% rate 170. While optimization to improve the delivery and editing efficiency of these systems is warranted before clinical application, their use to target tumors harboring mutant p53 may become potential therapeutic options in the foreseeable future.

#### Mutant p53 immunotherapies & immune checkpoint inhibitors

While mutant p53 can evade degradation by E3 ligases, it can still be degraded in MDM2-independent and proteasome-dependent pathways. Its peptides will eventually be presented on tumor cell surfaces by major histocompatibility complex (MHC) Class I. These serve as therapeutic targets for immunotherapies. The use of engineered T cell receptor-like antibody P1C1TM specific for a wild-type p53_125-134_ peptide in complex with HLA-A24 Class I MHC allele induces selective cytotoxicity and growth inhibition of mutant p53-expressing cells, including those of breast cancer cells, by distinguishing them based on their antigen expression levels 171. It is however crucial that future development of a similar antibody or any future chimeric antigen receptor T cells factors in the ability to recognize the subtle differences in the antigen expression between wild-type and mutant p53 cells to increase target specificity and minimize off-target effects.

Another immunotherapy strategy against mutant p53 is through vaccination of cancer patients using a wild-type human p53-expressing modified vaccinia Ankara virus (p53MVA). This strategy exploits the high expression of oncogenic mutant p53 protein as a target for the host immune system to mount a p53-specific cytotoxic T-lymphocyte response against mutant p53. Based on several Phase I trials, p53MVA vaccine is well-tolerated with no severe adverse events. Furthermore, it induces robust CD8+ T-cell p53 response in peripheral blood 172. The combination of the p53MVA vaccine with anti-PD-1 pembrolizumab in patients with advanced solid tumors including breast cancer resulted in disease control in 3/11 patients with 2 showing higher and more durable p53-reactive CD8+ T cells. 173. In a separate case study of a TNBC patient, this combination resulted in complete regression of metastasis, accompanied by activation of p53-specific T cell responses and immune response genes in peripheral blood 174. This suggests that vaccination against mutant p53 can be a viable option in breast cancer.

Vaccines can also be administered with a viral vector against mutant p53. An example is the Phase I/II study conducted using Gendicine and Indoximod in metastatic solid tumors and invasive breast cancer patients (NCT01042535). Gendicine was used to generate dendritic cell (DC) vaccine against p53 while Indoximod was used to enhance immunologic responses to DC vaccines. Immunologic responses were observed in 30.4% of evaluable patients and stable disease was observed in 4 out of 39 patients. 40.9% of patients also benefitted from chemotherapy after vaccination 175. In a similar trial, the use of autologous DC-adenovirus p53 vaccine was evaluated in Stage III breast cancer patients (NCT00082641). Patients received a total of 4 vaccinations and were stratified into two arms; Arm I received vaccination once after two different chemotherapy regimens and twice after radiotherapy & Arm II received 4 vaccinations after radiotherapy on separate weeks. All patients exhibited an immune response to the Gendicine DC vaccine in Arm I compared to 53% in Arm II. However, 45.5% of patients in Arm I developed serious toxicities compared to 8.3% in Arm II. These studies suggest that the use of Gendicine-based DC vaccines can be used in combination with chemotherapy to yield immunologic response against p53.

Recent data reveal associations between p53 and immunological checkpoints in cancer, and the status of p53 may dictate response to immune checkpoint inhibitors (ICIs) 176. It was reported that patients whose tumors harbored p53 missense mutations had increased tumor PD-L1 expressions as compared to those with wild-type and had a better response rate to anti-PD-1/PD-L1 177. ICIs also have the potential to reverse endocrine therapy resistance in luminal B breast cancer with high immunologic gene expressions 178. Despite this, the efficacy of ICIs as a monotherapy in breast cancer, especially in mutant p53-prevalent subtype like TNBC, is low. Thus, there is a need to develop new strategies to improve the clinical benefits of ICIs 179. Studies have shown that the use of p53 gene therapies resulted in increased tumor inflammation signatures and interferon signaling, along with increased CD8+ T cell signature that is associated with better clinical responses to ICIs 180, 181. Thus, ICIs have become a subject of several combinatorial regimens with various p53-specific targeted therapies. Examples include p53MVA 182, SGT-53 183, Ad-p53 184, Ad-p53 + CD122/132 181, OBP-702 (a wild-type p53 expressing oncolytic adenovirus), CXCR4-targeted p53 mRNA nanoparticles 185, and APR-246 with anti-PD-1 in several cancer models. While most of these combinations have not been evaluated in breast cancers, the consensus from these studies is that these various combinations resulted in 1) increased immune response such as cytotoxic T cell activities and CD8+ T cell infiltration, 2) a reduction in immunosuppressive cells (M2 macrophages) and expression of immunosuppressive regulators, and 3) have the potential to reverse resistance to ICIs. In July 2022, PMV Pharmaceuticals announced a clinical trial in collaboration with Merck evaluating the combination of PC14586 and pembrolizumab in advanced solid cancer patients harboring p53 Y220C mutation.

### Challenges in developing therapeutic strategies against p53 and future perspectives

As a target, p53 is a challenging protein to develop inhibitors against. This is attributed to it being a transcription factor that lacks accessibility of a receptor-ligand interaction or enzyme active site. Nonetheless, the understanding of p53 structure and its interaction partners has facilitated the discovery and development of many compounds that have the potential to restore the tumor suppressive activities of p53.

Many small molecule compounds with varying complexities have been designed to target both wild-type and mutant p53-bearing tumors, although most are still in pre-clinical stages of development 186. Only a handful of candidate drugs have been investigated in early phase clinical trials involving breast cancer cases (Table 3) and the results have been mixed with modest efficacy signals and high-grade toxicities, in contrast to their promising pre-clinical results. While the exact reasons for the difference in patient response to the small molecule inhibitors remains unknown, the unintended consequences of inhibiting a certain target associated with p53 should be factored into consideration. For example, in tumors with functional p53, the inhibition or knockout of MDM2 can trigger the activation of other oncogenic pathways 187. Additionally, targeting negative regulators of p53 may lead to widespread p53 activation that may affect healthy cells alike.

In the case of targeting mutant p53, the use of small molecule compounds for p53 reactivation is limited by the diverse set of mutations exhibiting varying structural alterations and molecular functions as none of the mentioned compounds in this review can target all p53 mutations. Furthermore, several of these strategies do not exclusively inhibit mutant p53 and may contribute to adverse events.

Another area of concern is intratumoral heterogeneity. Given the heterogeneous nature of breast tumors, the main question to be addressed is whether prolonged use of these compounds can exert a selection pressure of pre-existing resistant cells or give rise to *de novo* mutations that can limit their clinical benefits.

As most of the evaluated compounds have shown limited clinical efficacy as monotherapies, combination with other therapeutic agents is warranted. Moreover, there are several notable compounds that have other primary mechanisms of anti-cancer activities and have been evaluated in trials involving breast cancer patients, such as fulvestrant and several Hsp90 and HDAC inhibitors. While they exhibit secondary effects against either wild-type or mutant p53 models, their association with p53 mutational status has not been determined in patients. It may be of therapeutic value if future clinical trials are conducted taking into consideration the tumor's p53 mutational status to determine if such compounds have differential activities against wild-type versus mutant p53 tumors.

## Conclusion

The tumor suppressor p53 is a vital regulator of cellular responses. Both wild-type and mutant p53 protein are attractive therapeutic targets due to their distinct subtype-based prevalence in breast cancers. In this review, we have summarized the different therapeutic strategies that are being developed to target wild-type and mutant p53-bearing cells in breast cancer models. These strategies share a similar intended goal of minimizing the degradation of wild-type p53 and/or decreasing mutant p53 levels via chemical or genetic approaches.

In conclusion, p53 is a highly relevant therapeutic target for breast cancer and warrant further clinical development, especially in patients with the TNBC and HER2-positive subtypes with high p53 mutational burden. Optimization of p53-associated therapeutic strategies including rational combinations will be an important future direction.

## Figures and Tables

**Table 1:** Compounds against wild-type p53 tumors that have been evaluated in breast cancer models or patients

Compound name	Class/Derivatives	Breast cancer model validation	Stage of development	References
***MDM2-p53 inhibitors***				
Nutlin-1, 2, 3, 3a	Cis-imidazoline	ER+ (p53 WT), TNBC (p53 mutant), Numb-deficient breast PDXs	Pre-clinical	188-190
RG7112		ER+ (p53 WT)	Pre-clinical in breast cancer, Phase I/Ib clinical trial in solid tumors (NCT00559533) or hematological malignancies (NCT00623870)	191
Idasanutlin		ER+ (p53 WT), TNBC (p53 mutant)	Phase I/II clinical trial with atezolizumab in breast cancer (NCT03566485), up to Phase III clinical trial in AML (NCT02545283)	192
Compound 3		TNBC (p53 mutant)	Pre-clinical	193
Compound 5g	Spirooxindole	ER+ (p53 WT)	Pre-clinical	53
Compound 5d		ER+ (p53 WT), TNBC (p53 mutant)	Pre-clinical	57
Compound 5b		ER+ (p53 WT)	Pre-clinical	58
Compound 3d		ER+ (p53 WT)	Pre-clinical	59
MI-77301		Endocrine-resistant breast cancer PDXs (p53 WT)	Pre-clinical in breast cancer, Phase I clinical trial in solid tumors with Pimasertib (NCT01985191)	55
G613		ER+ (p53 WT)	Pre-clinical	56
AMG-232	Piperidinone	MDM2-overexpressing ER+ (p53 WT)	Phase I (NCT01723020)	65
CGM097	Dihydroisoquinolinone	ER+ cell lines (p53 WT & mutant) & ER+ PDX	Pre-clinical in breast cancer, Phase I clinical trial in solid tumors (NCT01760525)	64
Prenylchalcone 2e	Chalcone	ER+ (p53 WT)	Pre-clinical	62
Tricetin	Flavonoid	ER+ (p53 WT)	Pre-clinical	63
RITA (also inhibit MDMX and reactivate mutant p53)	Thiophene	ER+ (p53 WT)	Pre-clinical	61
***MDM2 inhibitors or degraders***				
Albendazole	Benzimidazole	ER+ (p53 WT)	Approved for general use, but not specifically developed in relation to MDM2	76
Fenbendazole				
Fulvestrant	Steroidal	ER+ (p53 WT & mutant)	Approved in breast cancer, but not specifically developed in relation to MDM2	77
SP-141	Pyrido[b]indole	ER+ (p53 WT and p53 knockdown), TNBC (p53 mutant)	Pre-clinical	74
CPI-7c	Pyrido[b]indole	ER+ (p53 WT)	Pre-clinical	75
JapA	Dimeric sesquiterpenoid	ER+ (p53 WT and p53 knockdown), TNBC (p53 mutant)	Pre-clinical	71
Inulanolide A			Pre-clinical	72
Lineariifolianoid A			Pre-clinical	73
Curcumin	Diarylheptanoid	ER+ (p53 WT)	Phase II clinical trial, but not specifically developed in relation to MDM2	78
Genistein	Isoflavone	ER+ (p53 WT)	Phase II clinical trial, but not specifically developed in relation to MDM2	79, 194
25-OCH3-PPD	Ginsenoside	ER+ (p53 WT), TNBC (p53 mutant)	Pre-clinical	80
Platycodin D	Triterpenoid	TNBC (p53 mutant)	Pre-clinical	81
FBA-TPQ, PEA-TPQ, MPA-TPQ, DPA-TPQ, BA-TPQ	Makaluvamine	ER+ (p53 WT), TNBC (p53 mutant)	Pre-clinical	82, 83
Melatonin	Acetamide	ER+ (p53 WT)	Phase I clinical trial in breast cancer, but not specifically developed in relation to MDM2	84
Gambogic acid	Xanthonoid	ER+ (p53 WT)	Phase IIa clinical trial in breast cancer, but not specifically developed in relation to MDM2	85
Gossypol	Phenolic aldehyde	ER+ (p53 WT & mutant), TNBC (p53 mutant)	Phase I/II clinical trial in breast cancer, but not specifically developed in relation to MDM2	86, 87
Hispolon	Hispolon	ER+ (p53 WT)	Pre-clinical	88
Oroxylin A	Flavonoid	TNBC (p53 mutant)	Pre-clinical	89
***MDMX inhibitors***				
XI-011, XI-006	Benzofuroxan	ER+ & HER2+ (p53 WT), TPBC & TNBC (p53 mutant)	Pre-clinical	49, 50
***Dual MDM2/MDMX inhibition***				
SAH-p53-8	Peptide	ER+ (p53 WT)	Pre-clinical	69
ATSP-7041	Peptide	ER+ (p53 WT)	Pre-clinical	70
ALRN-6924	Peptide	ER+ (p53 WT & mutant), HER2+ & TPBC (p53 mutant)	Phase Ib clinical trial with Paclitaxel in solid tumors with WT p53 including breast cancer (NCT03725436)	191
cTAT-KD3, cR10-KD3	Peptide	ER+ (p53 WT)	Pre-clinical	68
***Inhibitors of MDM2's E3 ligase activity***				
HLI98s, HLI373, HLI393	7-nitro-5-deazaflavin	TNBC (p53 mutant)	Pre-clinical	90, 91
JNJ26854165	Tryptamine derivative	-	Phase I clinical trial in solid tumors including breast cancer (NCT00676910)	93
Sempervirine	Indolo‐pyrido‐isoquinolin	ER+ (p53 WT)	Pre-clinical	92

ER+: estrogen receptor-positive; HER2+: human epidermal growth factor receptor 2-positive; PDX: patient-derived xenograft; TNBC: triple negative breast cancer; TPBC: triple positive breast cancer, WT: wild-type.

**Table 2:** Compounds and gene or immunotherapies against mutant p53 tumors that have been evaluated in breast cancer models or patients

Compound name	Mechanism of action	Stage of development	References
***Mutant p53 reactivators***			
PRIMA-1, APR-246	Michael addition; target cysteine residue to restore DNA binding ability of p53; prevent aggregation of mutant p53	Pre-clinical in breast cancer, up to Phase III clinical trial (APR-246 in combination with azacitidine) in myelodysplastic syndrome (NCT03745716)	100, 101, 195
PK11007	Selective alkylation of 2 cysteine residues, leading to increased thermal stability of DNA binding domain of p53 Y220C	Pre-clinical	104, 105
COTI-2	Zn^2+^ chelator, restore DNA binding ability of p53	Pre-clinical in breast cancer, Phase I clinical trial for several cancers (NCT02433626)	107
NSC319726 (or ZMC1)	Zn^2+^ chelator, restore DNA binding ability of p53	Pre-clinical	196
PC14586	Binds to crevices of Y220C mutant, leading to increased thermal stability of DNA binding domain of p53 Y220C	Phase I/II in advanced solid tumors, including breast cancer, with TP53 Y220C mutation (NCT04585750)	116
RITA	Not determined	Pre-clinical	197
KSS-9	Michael addition	Pre-clinical	109
R-goniothalamin	Michael addition	Pre-clinical	110
PEITC	Not determined	Pre-clinical	111
HO-3867	Michael addition	Pre-clinical	112
HBAP	Binds to DNA binding domain of p53 through hydroxyl group, restore transcriptional activity of wild-type p53	Pre-clinical	114
Chetomin	Binds to Hsp40, enhance binding to p53 R175H and restores wild-type-like function	Pre-clinical	113
***Mutant p53 degradation inducers***			
Hsp90 inhibitors (Geldanamycin, 17-AAG, Ganetespib)	Inhibits Hsp90, destabilizing mutant p53 and targeting it for degradation	Up to Phase II clinical trial in breast cancers, but not specifically developed against mutant p53 tumors	119, 198, 199
HDAC inhibitors (SAHA, NaB, Trichostatin A, sodium butyrate, and FR90122)	Inhibits HDAC6 which in turn inhibits Hsp90 to promote degradation; blocks transcription of mutant p53 gene	Up to Phase III clinical trial in breast cancers, but not specifically developed against mutant p53 tumors	119, 120
Arsenic trioxide	Induces mutant p53 proteasomal degradation; increases expression of E3 ligase, Pirh2	Phase II clinical trial in breast cancer, but not specifically developed against mutant p53 tumors	122, 200
Gambogic acid	Induces nuclear export of mutant p53 for CHIP-mediated degradation; prevents mutant p53-Hsp90 interaction	Pre-clinical	123
Spautin-1	Inhibits deubiquitinating proteins; promotes chaperone-mediated autophagy leading to lysosomal degradation of mutant p53	Pre-clinical	124
YK-3-237	Activates SIRT1 to reduce mutant p53 acetylation and its stability	Pre-clinical	125
Triptolide	Not determined	Pre-clinical	126
*Origanum majorana* extract	Not determined	Pre-clinical	127
THZ1	Inhibits CDK7 which is critical for mutant p53 expression	Pre-clinical	128
BEZ235	Not determined but degradation induction of mutant p53 is independent with the ubiquitin-proteasome pathway and autophagy	Up to Phase II clinical trial in breast cancer, but not specifically developed against mutant p53 tumors	129
CBISC	Not determined	Pre-clinical	130
Capsaicin	Not determined	Phase III clinical trial in breast cancer, but not specifically developed against mutant p53 tumors	131
Disulfiram	Induces glutathionylation of p53, targeting it for proteasomal degradation	Phase II clinical trial with copper in breast cancer, but not specifically developed against with mutant p53 tumors	132
ZIF-8 (and Z1-RGD)	Zn^2+^ chelator; increased mutant p53 gluthionylation	Pre-clinical	133
***Synthetic lethal inhibitors***			
MK-1775	Suppresses G2 arrest via Wee1 inhibition, leading to mitotic catastrophe	Up to Phase II clinical trial in breast cancer, but not specifically developed against with mutant p53 tumors	138, 139
Chk1 inhibitor (UCN-01, AZD7762)	Suppresses G2 arrest via Chk1 inhibition, leading to mitotic catastrophe	Up to Phase II clinical trial in breast cancer, but not specifically developed against mutant p53 tumors	141, 142
Onvansertib	Loss of G2/M checkpoint control by PLK1 inhibition	Pre-clinical in breast cancer, up to Phase II clinical trial in other cancers but not specifically developed against mutant p53 tumors	146
Roscovitine → Doxorubicin	Arrest cells at G2 with induction of DNA damage	Pre-clinical	143
Deoxyuridine analogues + PARP inhibitors	Arrest cells at G2 with induction of DNA damage	Pre-clinical	144
THZ-P1-2	Disrupts cell energy metabolism required for mutant p53	Pre-clinical	147
***Inhibitors of mutant p53 GOF interactors or pathways***			
Carfilzomib	Decreases protein turnover induced by mutant p53-Nrf2 interaction	Phase I clinical trial in breast cancer, but not specifically developed against mutant p53 tumors	149
Statins	Induce cytoplasmic relocalization of YAP/TAZ, leading to reduced sterol production that drives mutant p53 GOF activities	Up to Phase III clinical trial, but not specifically developed against mutant p53 tumors	150
GGTI-298		Pre-clinical	151
Zoledronic acid		Phase II clinical trials in breast cancer (NCT03358017 and NCT02347163)	
Pin1 inhibitors (ATRA & KPT-6566)	Disrupt mutant p53-Pin1 interaction that drives metastasis	Phase II clinical trial (ATRA) in breast cancer, but not specifically developed against mutant p53 tumors	152
PARP inhibitors (Olaparib, Talazoparib)	Negative regulation of DNA repair	Several PARP inhibitors (including Olaparib and Talazoparib) are approved in breast cancer, but were not specifically developed against mutant p53 tumors	33
GFP-KA2	Targets mutant p53-DAB2IP to reduce inflammation and inflammation-driven metastasis	Pre-clinical	155
COMPASS inhibitors	Inhibit downstream epigenetic effectors (COMPASS) of mutant p53 and ETS2 interaction	Pre-clinical	27
***Gene/Immunotherapies***			
Gendicine (Ad-p53)	Gene replacement	Phase I/II clinical trial (NCT00082641), with indoximod (NCT01042535), approved for head & neck cancer in China	157, 158, 175
Advexin (Ad5CMV-p53)		Phase II clinical trial with docetaxel and doxorubicin (NCT00044993)	159
ONYX-015	Selective replication and lysis of p53-null human tumor cells	Phase I clinical trial with etanercept	160
AdVING4/p53	Coexpression of ING4 and wild-type p53	Pre-clinical	161
Transferrin-SiNPs-p53	Deliver wild-type p53-carrying plasmid/ cDNA to tumor cells based on their higher transferrin receptors	Pre-clinical	162
SGT-53		Phase I clinical trial (monotherapy), Phase Ib clinical trial with docetaxel (NCT00470613)	165, 166
Ad-p53-CC	Inserting wild-type p53 gene that can overcome mutant p53 dominant negative effects	Pre-clinical	167, 168
P1C1TM	Induces antibody-dependent cellular cytotoxicity on mutant p53 based on antigen expression level	Pre-clinical	171
p53MVA	Induces p53-specific cytotoxic T-lymphocyte response to kill cells with mutant p53	Phase I clinical trial (NCT02432963) with pembrolizumab	172-174

ATRA: all-trans retinoic acid; CHIP: carboxy-terminus of Hsc70 interacting protein; COMPASS: complex protein associated with Set1; DAB2IP: DAB2 interacting protein; HDAC: histone deacetylase; Hsp: heat shock protein; ING4: inhibitor of growth 4; PARP: poly (ADP-ribose) polymerase; PLK1: polo-like kinase 1; SIRT1: sirtuin-1; YAP/TAZ: Yes-associated protein/ transcriptional coactivator with PDZ-binding motif

**Table 3:** List of clinical trials evaluating p53 targeted therapies that involve breast cancer patients in the past decade (2013-2023).

Compound	Intervention	Phase	Status	Conclusion	ClinicalTrial.gov identifier & reference
***Therapies against mutant p53 tumor***					
p53MVA	p53MVA (5.6 x 10^8^ pfu IM) Pembrolizumab (200 mg IV)	I	Active, not recruiting	3/11 with stable disease, 2 with increased and sustained T cell activity	NCT02432963 173, 174
SGT-53	SGT-53 (IV) on day 1, 8, 15 Pembrolizumab (IV) on day 3 Carboplatin (IV) on day 3	I	Withdrawn: funding and drug preparation issues	-	NCT05093387
Zoledronic acid	Zoledronic acid (4 mg IV) every 3 or 4 weeks Atorvastatin (80 mg oral) once daily	II	Unknown	-	NCT03358017
	Zoledronic acid (4 mg IV) 7 days before definitive breast surgery	II	Terminated: low accrual	-	NCT02347163
Ad-p53	Ad-p53 (dose according to tumor size; intratumoral) Clinician recommended ICI	II	Recruiting	Dose of Ad-p53 at above 7 x 10^10^ viral particles/cm^3^ of tumor corresponds to better response; improved immune activities	NCT03544723 180
PC14586	PC14586 (various doses oral)	I/II	Recruiting	Safe and well-tolerated up to 3000 mg daily; 1 breast cancer patient with partial response	NCT04585750 116
***Therapies against wild-type p53 tumor***					
Idasanutlin	Atezolizumab (840 mg IV) Idasanutlin (100 mg oral)	Ib	Terminated: low accrual/loss of funding	4 out of 7 patients with disease progression; serious adverse events	NCT03566485
AMG-232	AMG-232 (240 mg MTD oral)	I	Completed	7/12 ER+ patients with stable disease	NCT01723020 65
ALRN-6924	Paclitaxel (IV) ALRN-6924 (IV) on day 1, 8, 15	Ib	Recruiting	-	NCT03725436
	ALRN-6924 1.2 mg/kg (IV) Day 0-2 TAC: Doxorubicin 50 mg/m^2^ (IV); Cyclophosphamide 500 mg/m^2^ (IV); Docetaxel 75 mg/m^2^ (IV) Day 1 of every 3-week cycle	Ib	Recruiting	-	NCT05622058

ER+: estrogen receptor-positive; MTD: maximum tolerated dose; Pfu: plaque forming unit; ICI: immune checkpoint inhibitor; IM: intramuscular; IV: intravenous

## Abbreviations

ADORA2B: adenosine A2b receptor
AKT: Ak strain transforming
ATM: ataxia telangiectasia-mutated
ATR: ataxia telangiectasia and Rad3-related
ATRA: all-trans retinoic acid
BAX: Bcl-2-associated X
BC: breast cancer
BRCA: breast cancer gene
CDK: cyclin-dependent kinase
Chk1: checkpoint kinase 1
COMPASS: Complex proteins associated with Set1
CTD: C-terminal domain
DAB2IP: DAB2 interacting protein
DC: dendritic cell
DEPDC1: DEP domain containing 1
EGFR: epidermal growth factor receptor
ER: estrogen receptor
GOF: gain-of-function
HDAC: histone deacetylase
HER2: human epidermal growth factor receptor-2
HLA: human leukocyte antigen
Hsp: heat shock protein
ICIs: immune checkpoint inhibitors
IMiD: immunomodulatory drugs
ING4: inhibitor of growth 4
MDM2: murine double minute 2
MDMX: murine double minute X
MHC: major histocompatibility complex
MQ: methylene quinuclidinone
PARP: poly (ADP-ribose) polymerase
PD-1: programmed death-1
PD-L1: programmed death-ligand 1
PDX: patient-derived xenograft
PI5P4K: phosphatidylinositol 5-phosphate 4-kinase
PLK: polo-like kinase
PROTAC: proteolysis targeting chimera
PUMA: p53 upregulated modulator of apoptosis
Rb: retinoblastoma
RING: Really Interesting New Gene
ROS: reactive oxygen species
siRNA: small interfering RNA
SREBP: sterol regulatory-element binding protein
TNBC: triple negative breast cancer
TPBC: triple positive breast cancer
TRAIL: tumor necrosis factor-related apoptosis-inducing ligand
YAP/TAZ: Yes-associated protein/transcriptional coactivator with PDZ-binding motif
ZA: zoledronic acid
